# Weaning age influences indicators of rumen function and development in female Holstein calves

**DOI:** 10.1186/s12917-022-03163-1

**Published:** 2022-03-17

**Authors:** Sarah Schwarzkopf, Asako Kinoshita, Liane Hüther, Laurenz Salm, Saskia Kehraus, Karl-Heinz Südekum, Korinna Huber, Sven Dänicke, Jana Frahm

**Affiliations:** 1 Department of Functional Anatomy of Livestock, Institute of Animal Science, University of Hohenheim, Fruwithstr. 35, 70593 Stuttgart, Germany; 2 Institute of Animal Nutrition, Friedrich-Loeffler-Institute, Bundesallee 37, 38116 Braunschweig, Germany; 3 Institute of Animal Science, University of Bonn, Endenicher Allee 15, 53115 Bonn, Germany

**Keywords:** Weaning age, Calves, Rumen function and development, Acid–base balance, Parity

## Abstract

**Background:**

Prenatal and postnatal conditions are crucial for the development of calves. Primiparous cows are still maturing during pregnancy, thus competing with the nutritional needs of their offspring. Therefore, mature cows might provide a superior intrauterine condition. Furthermore, weaning calves at an older age might affect them positively as well by reducing stress and offering time for various organs and their functions to develop. We aimed to evaluate effects of mothers’ parity and calves’ weaning age on gastrointestinal development and corresponding acid–base balance. Fifty-nine female German Holstein calves (about 8 days old) were investigated in a 2 × 2 factorial experiment with factors weaning age (7 vs. 17 weeks) and parity of mother (primiparous vs. multiparous). Calves were randomly assigned to one of these four groups. Animal behavior that was observed included resting, chewing and active behavior.

**Results:**

Behavioral patterns were interactively affected by time and weaning age. Rumen sounds per 2 min increased in early-weaned calves during their weaning period. In late-weaned calves a consistently increase in rumen sounds was already recorded before their weaning period. Urinary N-containing compounds (creatinine, hippuric acid, uric acid, urea, allantoin) were interactively affected by time and weaning age. Concentrations of all measured compounds except urea increased during early weaning. All except hippuric acid concentration decreased in early-weaned calves after weaning. In late-weaned calves allantoin and uric acid increased before weaning and did not change during weaning.

**Conclusion:**

These results suggest that late-weaned calves developed adequate rumen functions and acid–base balance, whereas early-weaned calves might have suffered from ruminal acidosis and catabolism. Weaning calves at 7 weeks of age might be too early for an adequate rumen development.

## Background

The prenatal and early postnatal periods are critical times of development. Any intervention may cause life-long changes in metabolism, which is termed metabolic imprinting, and therefore have a great impact on health and performance in later life [1]. The parity of the mother could have a major impact on her calf during the prenatal period [2]. Multiparous cows need to share energy and nutrients between the fetus and the lactating mammary gland, and primiparous cows are still maturing during pregnancy, thereby competing with the needs of their offspring. Both conditions could lead to a nutritional restriction of the calf. Calves are born as non-ruminant animals, which rely mainly on lactose and milk fat as energy supply. At weaning, the dependence on rumen fermentation increases and volatile fatty acids (VFA) become the most important source of energy [3]. This implies great modifications of digestive physiology. Rumen weight and epithelial area increased when liquid feed was replaced by solid feed at early age [4]. Weaning also changed the expression of genes involved in the cell cycle, lipid metabolism, molecular transport, cell morphology and death, cellular growth, and proliferation in the rumen epithelium [5]. Therefore, these indicators of a rapid rumen development appeared to be induced by a forced change from liquid to solid feed. On the contrary, other indicators were not influenced by weaning. In lambs, the expression of ketogenic enzymes in rumen epithelium increased with age, regardless of diet and time of weaning [6]. Therefore, rumen development might at least partly be controlled by an evolutionary blueprint. The growth of different gastrointestinal compartments is usually not finished at weaning. The weight of the reticulorumen and omasum relative to body weight (BW) increased until 17 weeks (wk) of age [7]. The omasum kept growing until 1 year of age [3]. Consequently, early weaning might not be necessary for rumen development. As a hypothesis, gastrointestinal development still proceeds when weaning is delayed. Late weaning may minimise the abrupt changes associated with solid feed intake and related metabolic changes, which occur during early weaning. Parity of the mother might impact their calves’ development through potential restriction of resources as well. Therefore, the present study aimed to determine the influence of mothers’ parity and calves’ weaning age on gastrointestinal tract development and associated systemic metabolic changes such as acid base homeostasis and early N metabolism. Transition was assessed by markers of ruminal development, and of corresponding adaptive changes in acid–base balance and gut microbial metabolism.

## Results

### Characterization of feed intake pattern

All data according to feed intake was previously published in Schwarzkopf et al. [8]. In summary, the intake pattern of milk replacer (MR) and concentrate (C) was similar for both weaning groups until experimental day 28 when weaning was initiated for early-weaned group and there were no significant differences in intake levels (122 – 133 g DM/day). While the late-weaned calves were maintained unchanged at a constant MR level (approximately 1300 g DM/day) and C intake continued to increase until reaching 2 kg on day ~ 70, MR intake for the early-weaned group was gradually reduced until day 42 of the experimental trial. At this time, C intake was similar to the late-weaned group. On experimental days 70 and 98, the early-weaned group was characterized by a TMR feeding pattern while the late-weaned group still consumed significant amount of MR. On experimental day 112, it is assumed that the late-weaned group consumed greater amounts of roughage as MR intake was terminated. At the same time, C intake was reduced to 1 kg/day, so that the general ration type was roughly comparable to that of the early-weaned group. On experimental day 140, all groups received the same ration type.

### Animal behavior

**Fig. 1:**
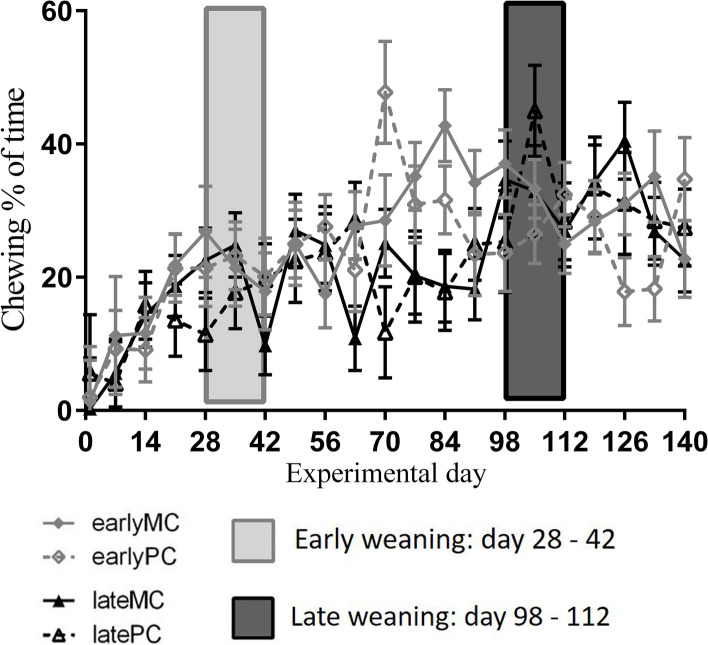
Percentage of time spent with chewing. Early-weaned calves were weaned gradually between experimental d 28 and 42 of the trial. Late-weaned calves were weaned gradually between experimental d 98 and 112 of trial. Data shown as LSMeans with SE. Early-weaned calves from multiparous cows (earlyMC), late-weaned calves from multiparous cows (lateMC), early-weaned calves from primiparous cows (earlyPC), late-weaned calves from primiparous cows (latePC)

**Fig. 2:**
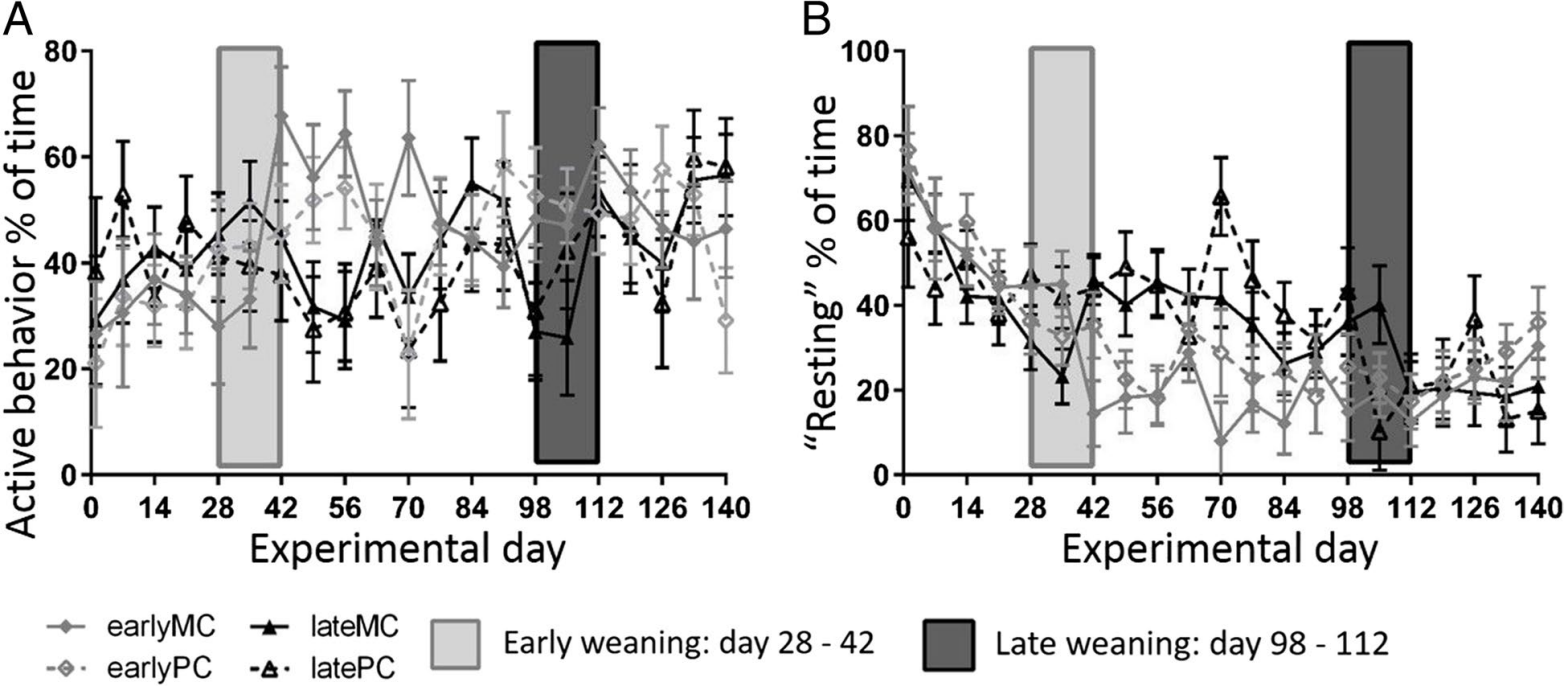
Percentage of time spent with active behavior (**A**) and resting (**B**). Early-weaned calves were weaned gradually between experimental d 28 and 42 of the trial. Late-weaned calves were weaned gradually between experimental d 98 and 112 of trial. Data shown as LSMeans with SE. Early-weaned calves from multiparous cows (earlyMC), late-weaned calves from multiparous cows (lateMC), early-weaned calves from primiparous cows (earlyPC), late-weaned calves from primiparous cows (latePC)

All observed behavior patterns changed over time and were interactively influenced by time and weaning age (Table 2). Percentage of time spent with chewing increased with age (Fig. 1). Early-weaned calves increased chewing at d 70 of the trial compared with d 1 (*p* < 0.001). Late-weaned calves increased their time spent with chewing at d 105 of the trial compared to d 1 (*p* = 0.002). Time spent with chewing exceeded 20% after d 21 (earlyMC and earlyPC), 28 (lateMC) or 49 (latePC). Time spent with active behavior increased over time (Fig. 2 A). There was, however, neither a significant difference between the weaning groups on any experimental d nor between the d in each weaning group. Time spent with resting decreased with age in both groups (Fig. 2 B), and the decrease was more pronounced in early-weaned calves. These spent significantly less time resting on d 35 compared to d 1 (*p* = 0.016), whereas late-weaned calves did not decrease the percentage of time spent resting until d 112 (*p* = 0.005).

### Rumen fill and sound

**Fig. 3:**
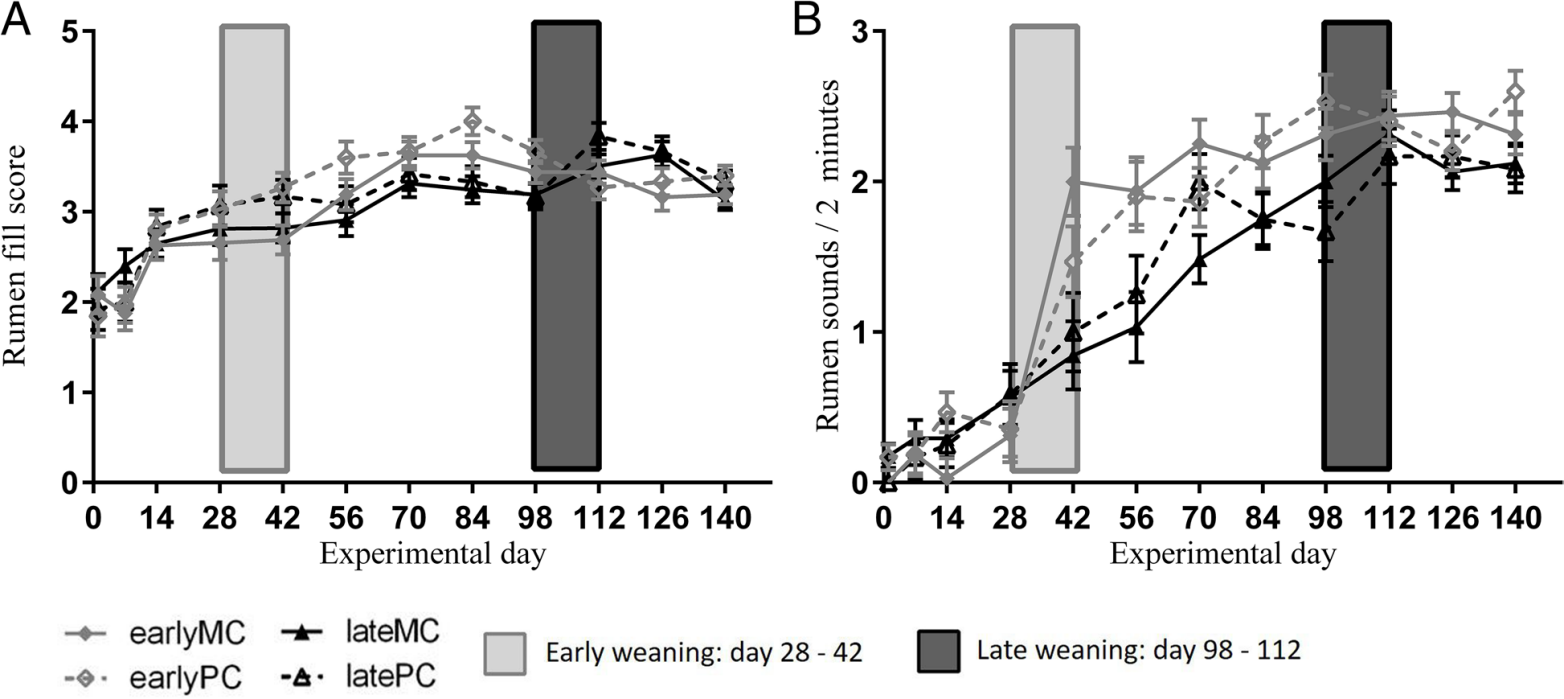
Rumen fill score (**A**) and sounds (**B**). Early-weaned calves were weaned gradually between experimental d 28 and 42 of the trial. Late-weaned calves were weaned gradually between experimental d 98 and 112 of trial. Data shown as LSMeans with SE. Early-weaned calves from multiparous cows (earlyMC) *n* = 16, late-weaned calves from multiparous cows (lateMC) *n* = 16, early-weaned calves from primiparous cows (earlyPC) *n* = 15, late-weaned calves from primiparous cows (latePC) *n* = 12. P < 0.05 was set as the level of significance.

Rumen fill (Fig. 3 A) and sounds (per 2 min, Fig. 3 B) increased significantly over time. A significant interaction between time and weaning age was observed for both variables (Table 2). Interaction between time, weaning group and parity was significant for rumen sounds. Rumen sounds increased during early weaning (d 28 vs. d 42; *p* < 0.001), whereas in late-weaned calves it increased already before weaning (latePC d 1 vs. d 56, *p* = 0.003; lateMC d 1 vs. d 70, *p* < 0.001) and therefore weaning caused no further increase (d 98 vs. d 112, *p* = 0.804). Rumen fill increased from d 1 to d 14 in both weaning groups (early *p* < 0.001; late *p* = 0.003). It was also influenced by parity (Table 2) with PC yielding a greater score. At the end of trial, there were no significant differences between weaning groups regarding rumen fill and sounds. The VFA and ammonia-N concentrations and pH in ruminal fluid at d 140 showed no effects of weaning age, mother’s parity and their interaction (Table 1).

**Table 1:** Effects of weaning age and parity on ammonia, VFA, and pH in rumen fluid evaluated by two-way ANOVA on experimental d 140. Values are presented as Means with SD

	Early-weaning		Late-weaning		*p*-values		
Parameters	MC	PC	MC	PC	Weaning age W	Parity P	W*P
Ammonia-N (mg/100 g)	2.33 ± 1.25	1.91 ± 1.71	2.93 ± 2.07	2.52 ± 1.18	0.144	0.289	1
Acetate (mmol/L)	35.89 ± 6.71	37.99 ± 8.23	36.15 ± 6.78	38.06 ± 5.88	0.971	0.278	0.917
Propionate (mmol/L)	9.44 ± 1.91	10.42 ± 2.18	9.91 ± 2.38	10.65 ± 2.89	0.605	0.170	0.723
Butyrate (mmol/L)	5.13 ± 1.67	5.76 ± 1.67	5.11 ± 1.50	5.52 ± 2.00	0.741	0.234	0.876
Iso-Butyrate (mmol/L)	0.66 ± 0.15	0.74 ± 0.16	0.67 ± 0.15	0.74 ± 0.20	0.948	0.099	0.974
Valerate (mmol/L)	0.70 ± 0.19	0.82 ± 0.21	0.74 ± 0.24	0.72 ± 0.22	0.630	0.337	0.238
Iso-Valerate (mmol/L)	1.10 ± 0.52	1.36 ± 0.55	0.99 ± 0.22	1.15 ± 0.46	0.172	0.078	0.665
Total VFA (mmol/L)	52.92 ± 10.25	57.10 ± 12.12	53.57 ± 10.23	56.84 ± 10.22	0.990	0.189	0.856
Ratio acetate:propionate	3.84 ± 0.47	3.67 ± 0.44	3.74 ± 0.60	3.69 ± 0.56	0.781	0.435	0.643
pH	7.34 ± 0.21	7.32 ± 0.27	7.35 ± 0.21	7.32 ± 0.17	0.857	0.684	0.960

**Table 2:** Effects of time, mother's parity and weaning age and their interaction on parameters shown in Figs. 1, 2, 3, 4, 5 and 6

*p*-Values							
Parameter	Time (T)	Parity (P)	Weaning age (W)	T x P	T x W	P x W	T x P x W
Chewing	< 0.001	0.440	0.042	0.512	0.016	0.525	0.277
Active behavior	0.037	0.308	0.085	0.750	0.001	0.850	0.803
Resting	< 0.001	0.110	0.004	0.575	< 0.001	0.548	0.442
Rumen fill score	< 0.001	0.018	0.490	0.335	0.001	0.455	0.780
Rumen sounds	< 0.001	0.77	< 0.001	0.965	< 0.001	0.938	0.042
pH saliva	< 0.001	0.638	0.295	0.621	0.674	0.171	0.299
pH feces	< 0.001	0.994	0.155	0.891	0.004	0.047	0.827
pH urine	< 0.001	0.525	0.069	0.652	< 0.001	0.670	0.773
pH blood	< 0.001	0.654	0.206	0.172	0.010	0.290	0.716
Creatinine	< 0.001	0.253	0.628	0.125	< 0.001	0.239	0.544
Allantoin	< 0.001	0.152	0.274	0.126	< 0.001	0.684	0.646
Uric acid	< 0.001	0.057	0.152	0.075	< 0.001	0.431	0.387
Urea	< 0.001	0.815	0.625	0.161	0.005	0.539	0.643
NABE	< 0.001	0.523	0.063	0.411	< 0.001	0.584	0.329
BAR	< 0.001	0.712	0.024	0.528	0.005	0.835	0.312

### pH in saliva, feces, and blood

The pH value in saliva increased over time and was not affected by weaning age or mother’s parity (Table 2). It increased from d 1 to d 42 (*p* < 0.001) and then from d 42 to d 140 (*p* = 0.011). Interaction between time and weaning age was significant for pH of feces (Table 2). It increased similarly in all groups from experimental d 1 to d 28 (*p* < 0.001). The highest pH values were observed at d 28 of the trial with no significant differences between the groups (earlyMC pH = 7.25; latePC pH = 6.99; lateMC pH = 7.06; earlyPC pH = 7.18). Afterwards, it decreased over time. The steepest pH decrease was observed during weaning in both weaning groups (early-weaned d 28 vs. d 42, *p* = 0.002; late-weaned d 98 vs. d 112, *p* = 0.018). Hence, it was different between weaning groups on experimental d 98 (*p* = 0.043). However, late-weaned calves had a significant decrease in feces pH before weaning (d 28 vs. d 70, *p* = 0.044).

**Fig. 4:**
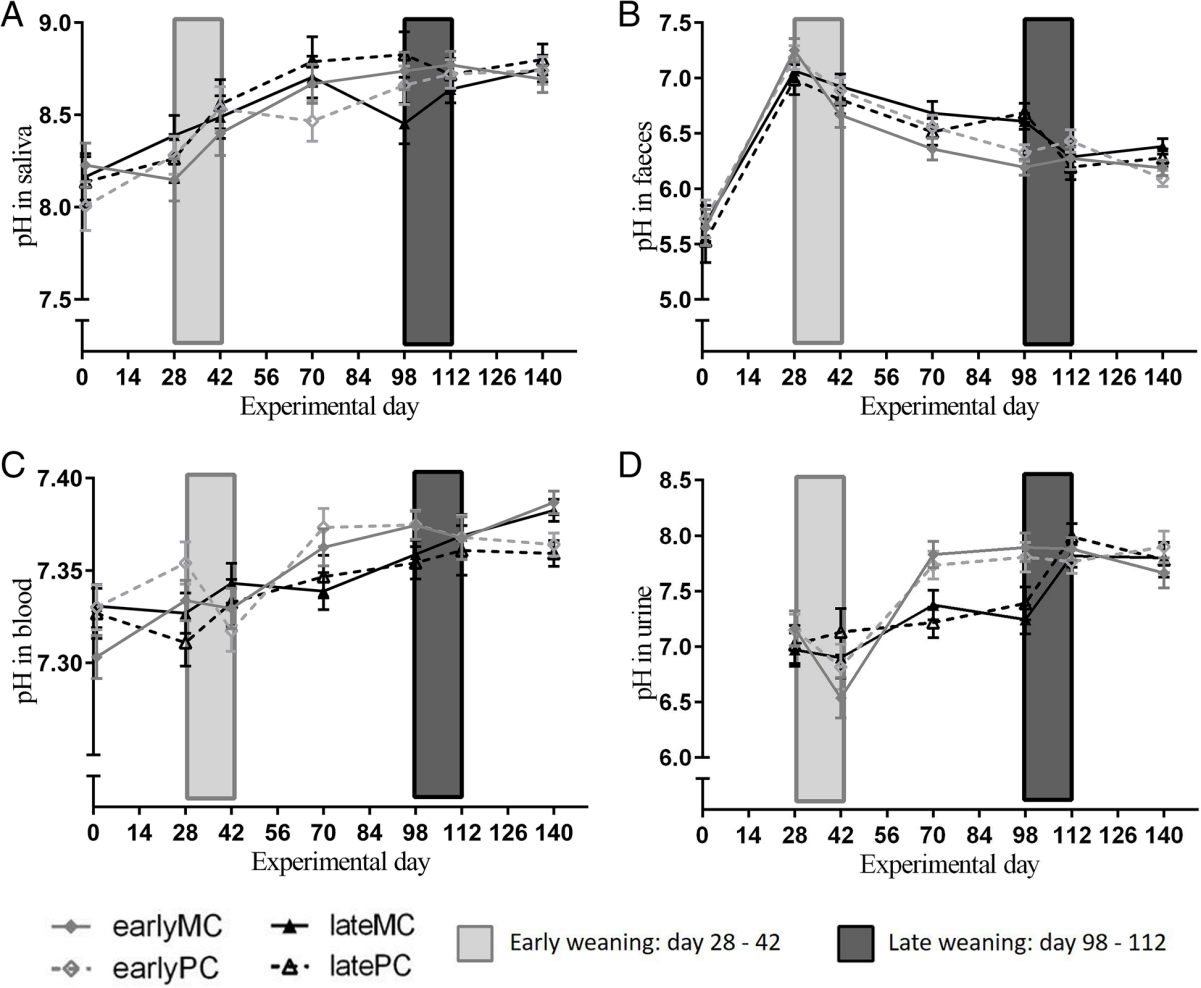
PH values of saliva (**A**), faeces (**B**), blood (**C**) and urine (**D**). Early-weaned calves were weaned gradually between experimental d 28 and 42 of the trial. Late-weaned calves were weaned gradually between experimental d 98 and 112 of trial. Data shown as LSMeans with SE. Early-weaned calves from multiparous cows (earlyMC) *n* = 16, late-weaned calves from multiparous cows (lateMC) *n* = 16, early-weaned calves from primiparous cows (earlyPC) *n* = 15, late-weaned calves from primiparous cows (latePC) *n* = 12

Whole blood pH increased over time (Fig. 4 C) and was also interactively influenced by time and weaning age, but it was not significantly different between groups at any sampling d. It increased from experimental d 42 to d 70 in early-weaned calves (*p* < 0.001).

### Variables in urine

The pH value in urine was interactively affected by time and weaning age (Table 2). During early weaning, it decreased (d 28 vs. d 42, *p* = 0.010) and then increased within the experimental period from d 42 to d 70 (*p* < 0.001). Afterwards, it remained stable for the rest of the experimental time. For late-weaned calves urinary pH stayed low until d 98 (d 28 vs. d 98, *p* = 0.624) and then increased during weaning (d 98 vs. d 112, *p* = 0.002) without dropping down before.

Specific density of urine was interactively influenced by time and weaning age (*p* < 0.001; data not shown). It increased in early-weaned calves between d 28 and 42 (*p* < 0.001). Therefore, it was higher in early-weaned calves than in late-weaned calves on experimental d 42 (*p* = 0.005). In late-weaned calves, it increased until d 70 (*p* < 0.001) and then remained stable on this level. Furthermore, specific density did not change through weaning in late-weaned calves (*p* = 0.1).

**Fig. 5:**
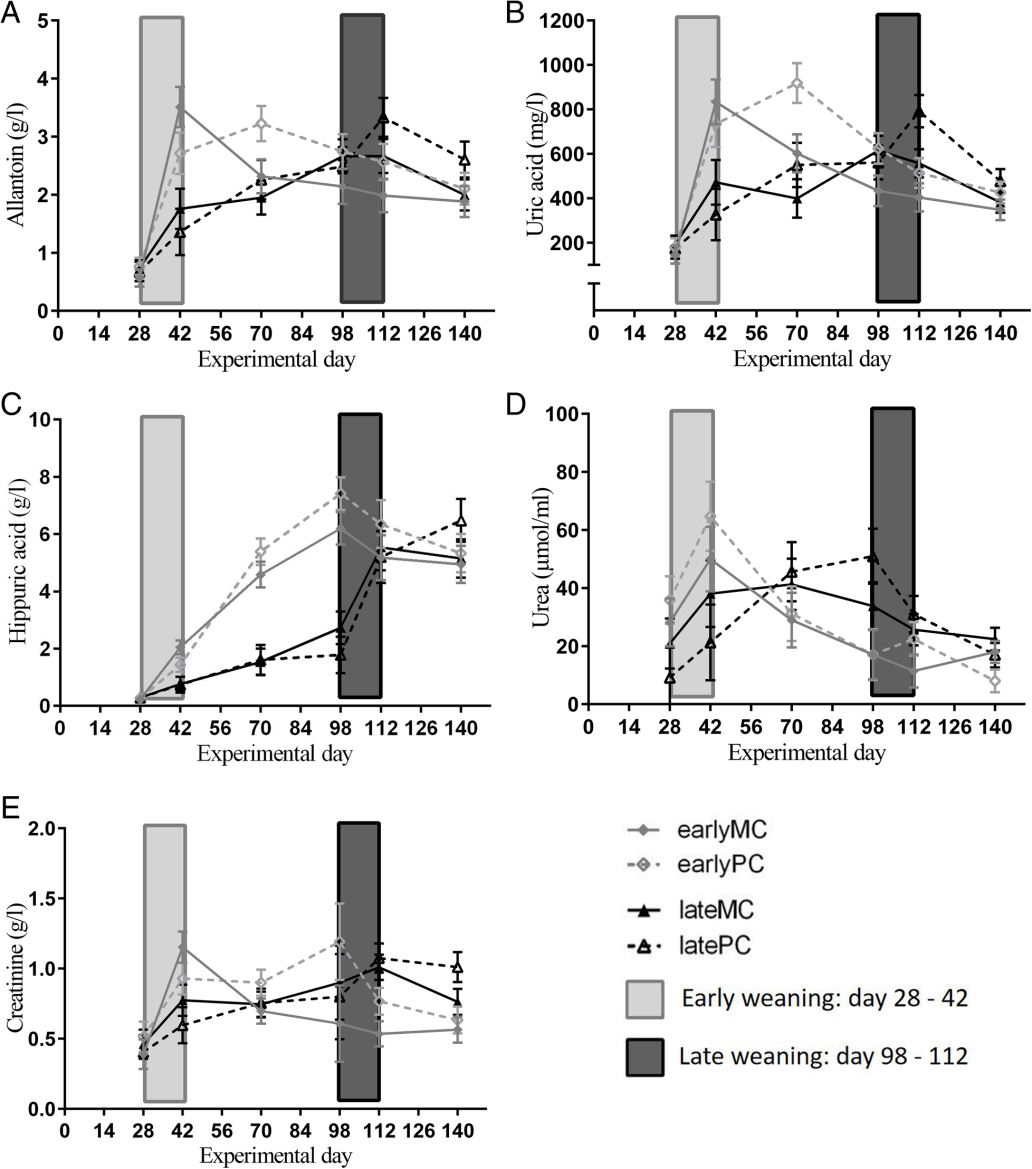
Concentrations of nitrogen-containing compounds in urine. Early-weaned calves were weaned gradually between experimental d 28 and 42 of the trial. Late-weaned calves were weaned gradually between experimental d 98 and 112 of trial. Data shown as LSMeans with SE. Early-weaned calves from multiparous cows (earlyMC) *n* = 16, late-weaned calves from multiparous cows (lateMC) *n* = 16, early-weaned calves from primiparous cows (earlyPC) *n* = 15, late-weaned calves from heifers (lateHC) *n* = 12

Nitrogen-containing compounds in urine (creatinine, hippuric acid, uric acid, urea and allantoin) were interactively affected by time and weaning age (Table 2). Concentrations of all compounds except urea increased during weaning in early-weaned calves (d 28 vs. d 42; *p* < 0.001). Urinary allantoin (*p* = 0.004; Fig. 5 A), uric acid (*p* = 0.027; Fig. 5 B) and hippuric acid (*p* = 0.016; Fig. 5 C) were higher in early-weaned calves on d 42, when they were weaned, compared to late-weaned calves that still received MR. Nitrogen-containing compound concentrations decreased in early-weaned calves after weaning except hippuric acid concentration. The urinary urea concentration decreased from d 42 to d 98 (*p* = 0.004; Fig. 5 D). Urinary creatinine (Fig. 5 E) and uric acid (Fig. 5 B) concentrations decreased from experimental d 42 to d 112 (*p* < 0.01). Urinary allantoin concentration was lower at the end of the experimental period (d 140) compared to the d after weaning (d 42; *p* = 0.019). Urinary hippuric acid concentration increased in early-weaned calves until d 70 (d 28 vs. d 70 *p* < 0.001) and did not change significantly thereafter. It also increased in late-weaned calves form d 28 to d 70 (*p* = 0.020), while the steepest increase occurred during weaning (*p* = 0.004; Fig. 5 C). All other N-containing compound concentrations did not change during weaning in late-weaned calves. Urinary creatinine concentration of late-weaned calves increased over time from experimental d 28 to 112 (*p* < 0.001). The urinary allantoin concentration increased before weaning (d 28 vs. d 70 *p* < 0.001). Urea concentration did not change significantly during weaning in both weaning groups and was not different between the groups on any experimental d.

**Fig. 6:**
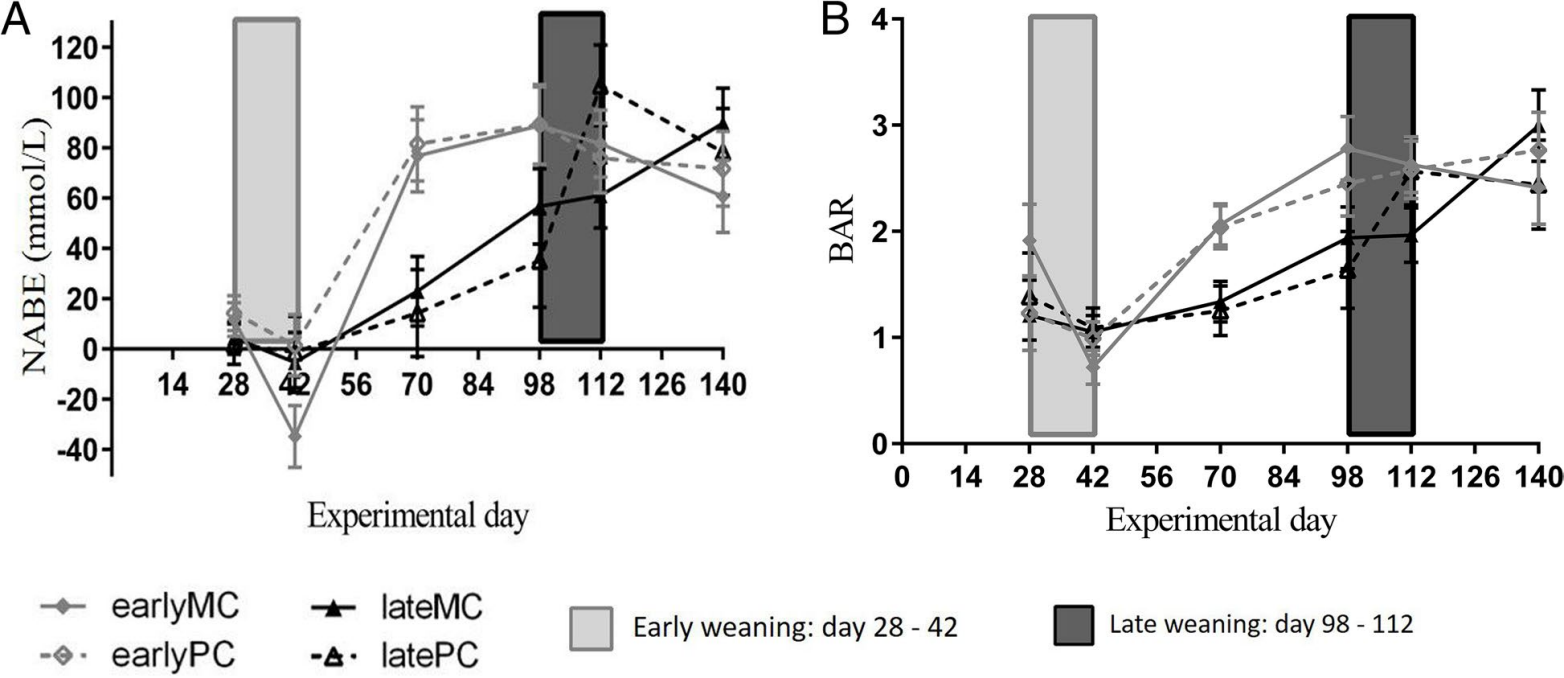
Values of net acid base excretion (NABE) (**A**) and base-acid ratio (BAR) (**B**) in urine. Early-weaned calves were weaned gradually between experimental d 28 and 42 of the trial. Late-weaned calves were weaned gradually between experimental d 98 and 112 of trial. Data shown as LSMeans with SE. Early-weaned calves from multiparous cows (earlyMC) *n* = 16, late-weaned calves from multiparous cows (lateMC) *n* = 16, early-weaned calves from primiparous cows (earlyPC) *n* = 15, late-weaned calves from heifers (lateHC) *n *= 12

All variables of urine were standardized to the corresponding specific density (data not shown), but no differences to unstandardized data regarding statistical data evaluation were observed. The NABE (*p* < 0.001; Fig. 6 A) and BAR (*p* = 0.005; Fig. 6 B) values in urine were interactively affected by time and weaning age (Table 2). Both increased after weaning in early-weaned calves (d 42 vs. d 70 *p* < 0.001). Therefore, they were higher in these than in late-weaned calves on d 70 (NABE *p* = 0.010; BAR *p* = 0.027). NABE values increased in late-weaned calves before weaning (d 42 vs. d 98, *p* = 0.030).

## Discussion

Studying weaning at different ages is challenging for research approaches. The regimen of weaning at younger age has different requirements compared to weaning at older age. In this study, for instance, calves at 15 wk of age voluntarily ingest more concentrate than calves at 5 wk of age (8). To provoke an increase in concentrate intake at this early age, MR consumption has to be reduced. As a result, concentrate intake increased to 1 kg/d. In calves at 15 wk of age, the concentrate intake was twice as high compared to the early-weaned calves. Thus, at the beginning of weaning, the concentrate intake was limited to 1 kg/d in the late-weaned group. After weaning, all calves received hay and a TMR. The effects of two different rearing regimens and mother’s parity are discussed in the following.

Rumen maturation at early age was assessed by behavioral observations, rumen sounds and scoring of rumen fill. Closely related to rumen maturation, acid base homeostasis and N metabolism have to adapt to ruminant status. Thus, the pH in blood, urine, feces and saliva was measured to assess changes in systemic acid–base-metabolism going along with rumen maturation. Furthermore, the concentration of several N-containing compounds (creatinine, allantoin, uric acid, hippuric acid and urea) in urine was determined to estimate the onset of microbial activity in the gastrointestinal tract, especially in the rumen, and to assess the development of early N metabolism in growing calves.

Mother’s parity had no effect on any parameter except rumen fill score (*p* = 0.018). This indicated that rearing conditions have a greater impact on rumen development, maturation of acid–base homeostasis and N metabolism than mother’s parity. Rumen fill score was greater in PCs, which might be caused by their smaller body seize paired with the same feed intake as MCs.

### Rumen maturation at early age

Late-weaned calves increased their solid feed intake before weaning [8]. As a consequence, rumen fill score and rumen sound increased before weaning as well. Rumen sounds per 2 min were assessed to get insights into progress of rumen motility, as it is possible to notice the sound of ingesta movements in the rumen with a stethoscope. Biphasic contractions of the reticulum were observed in milk-fed calves at the age of 16 d through ultrasonographic examinations [9]. In the current study, some calves had detectable rumen sounds as early as during the first wk of the trial, that corresponds to an age of ~ 14 days. This indicated that rumen sound and therefore rumen contraction were not only influenced by solid feed intake, but might be determined, at least partly, by an evolutionary blueprint. This might indicate that the nuclear genome was partly determining the age and velocity of maturation of organs and tissues of newborn and young calves with regard to their structure and function. Adverse nutritional interventions, especially during this fragile time window, could deteriorate this process leading to metabolic stress due to immature organs and tissues.

Confirming this assumption, early-weaned calves belatedly increased their time spent chewing from experimental d 70 on, which was 4 wk after weaning. This happened despite the ad libitum supply of hay and TMR, which is as solid feed known to increase rumination. However, late-weaned calves increased chewing during weaning at experimental d 105 already. The immature rumen might have contributed to lower solid feed intake in early-weaned calves. Thus, these calves were undernourished over several wk which was reflected in a lower live weight gain [8]. Concomitantly, early-weaned calves expressed a more active behavior after weaning up to experimental d 70, most likely due to seeking for liquid feed. In late-weaned calves, weaning was not associated with higher activity levels afterwards.

Calves that could freely access different solid feed and MR spent 20% of time with chewing both at the age of 3 and 6 mo [10]. As these calves displayed a low level of non-nutritive oral behavior, such as tongue playing and oral manipulation of the pen structure or other calves, a chewing level of 20% appeared to be enough to satisfy needs for chewing and rumination. This level was reached by our feeding groups at d 21 (earlyMC and earlyPC), 28 (lateMC) and 49 of the trial (latePC) and remained stable or increased most of the time (Fig. 1). Therefore, weaning at 17 wk of age may not trigger abnormal behaviour. The results of the aforementioned and the current study were similar, although we observed behavior only in a small time frame during the day because chewing was found to have no circadian rhythm in calves [11].

### Maturation of acid–base homeostasis in growing calves

Maturation of the gastrointestinal tract might be associated with maturation of systemic acid–base homeostasis. To assess its development, the pH values in saliva, blood, urine, and feces were measured during early life in all calves. As pH in saliva increased over time, being unaffected by weaning age or mothers’ parity, it seemed to be determined solely by the evolutionary blueprint. The first significant increase in pH compared to experimental d 1 was observed at d 42 (*p* < 0.001) and then again from d 42 to d 140 (*p* = 0.01) in all groups. Therefore, there might be an ontogenetic window for development of ruminant salivary buffer composition during these time points. The salivary gland responsible for secreting large amounts of buffer in ruminants is the *Glandula parotis*. The main chemical components of *Parotis* saliva are Ca^2+^, Na^+^, K^+^, Urea, HCO_3_^−^, HPO_4_^2−^, Cl^−^ and water. In general, the concentration of these components did not vary much between healthy adult cows and were unaffected by diet. Only urea concentration was affected by diet in adult cows [12]. The saliva buffering capacity and alkaline pH is mainly achieved by HCO_3_^−^ and HPO_4_^2−^ [13]. Therefore, these components are vital for an adequate rumen function. The change in salivary pH indicated that the adequate composition needed time to develop. However, the capacity of salivary glands, especially of the *Parotis*, to produce adequate volumes of saliva to buffer VFA production in the rumen was not assessed in this study.

As a consequence of the associated slow rise of salivary buffer capacity, early weaning, particularly before the age of 7 wk, could lead to acidification of rumen fluid. Ingestion of starch-rich concentrate might have increased rumen VFA concentration and thereby, decreased rumen pH value. This possible acidotic condition was most likely not balanced by a sufficient availability of saliva, due to low buffer concentrations and saliva volume, respectively. Additionally, absorption of VFA by the rumen epithelium might not be expressed with a sufficient capacity leading to an accumulation of acids in the rumen at this age. Furthermore, chewing activity, which is strongly influencing saliva secretion rate, is low after weaning in early-weaned calves. Others noticed different rumination patterns attributable to weaning age as well. Calves weaned at the age of 8 wk displayed more rumination before weaning than calves weaned at the age of 6 wk [14]. Therefore, it was hypothesized that early-weaned calves were at risk for ruminal acidosis, and for higher acid load in plasma as well. Several authors observed an acidic ruminal pH in early-weaned calves [14, 15]. Calves that were weaned at 4 wk of age had a ruminal pH under 5.5 for at least 4 wk after weaning (15). Weaning at 6 wk of age reduced ruminal pH below 5.5 at least for 3 wk as well [14]. As this might be a sign for chronic rumen acidosis at least for adult cows [16], it is possible that these calves suffered from this disease as well.

To maintain plasma pH is vital, thus, the lung and the kidneys are important regulators of acid–base homeostasis. While in the lung protons were eliminated as CO_2_ and H_2_O, kidneys excrete protons or reabsorb bases to maintain acid–base balance. Physiologically, urinary pH of healthy fully ruminating cattle is neutral to slightly alkaline [17, 18] due to the high amounts of excreted bicarbonate in herbivores. In the condition of metabolic acidosis, the kidney conserved bicarbonate by reabsorption [19]. As a consequence, the urinary pH value declined with decreasing bicarbonate concentration [20]. Consequentially, urinary pH value correlated positively with ruminal pH value [16]. Therefore, urinary pH might reflect the ruminal pH value and the decrease of urinary pH during early-weaning might have indicated rumen acidosis during this time. In early-weaned calves a potentially higher acid load needed more bicarbonate, thus this buffer was reabsorbed in the kidney. Consequently, pH of urine decreased (experimental d 42, 6.6 ± 0.1) directly after early-weaning (Fig. 4D). Furthermore, a higher NH_4_^+^ and phosphate concentration may also be responsible for the lower urinary pH value after early weaning, which might result from still low utilization of N-containing compounds in rumen microbial metabolism.

Furthermore, low urinary pH value was associated with greater calcium excretion, probably bound to acid phosphate [21]. As calcium is important for growth and bone development, this could have contributed to the impaired growth of early-weaned calves [8].

In adult cows, values of NABE and BAR in urine were used to diagnose a mild metabolic acidotic burden [22]. In calves this might not be advisable, as both variables are low in milk-fed calves (Fig. 6). The values of NABE and BAR increased over time, which reflected the transient change in dietary composition. In early-weaned calves, the increase was steep after weaning from experimental d 42 until d 70, but not during their weaning. The metabolic adaptation to an adult status seemed to occur after weaning was already done. On the other hand, the increase of NABE values in late-weaned calves was not as steep but occurred before weaning. They might have experienced a smoother transition to adult status.

The pH value in feces decreased during weaning (Fig. 4 B). Lohakare et al. [23] measured a pH of 7 in feces of calves at 40—42 d of age. Confirming, in the current study calves were 37 ± 2 d of age at experimental d 28 and had a faecal pH around 7 as well. Lohakare et al. [23] observed a decrease of faecal pH through weaning as well, but this increased again to a pH of 6.8 – 7.0 at 42 d after weaning. This increase was not detected in the current study, in which faecal pH continued to decline. Adult cattle had a faecal pH from 7 – 8 [24, 25], which, in this study, was not reached by any group until the end of the trial. This may be a result of high intake of starch through concentrate and TMR which could not be digested in rumen and therefore was transported into the large intestine. In the hindgut, it could be fermented, but emerging VFA could not be fully absorbed, subsequently leading to a decrease in pH value. The decrease in faecal pH through increased energy intake and carbohydrate infusion has been tested in steers [25], as well as a strong correlation between faecal starch content and pH [24]. Calves had a higher percentage of faecal starch after weaning, especially when weaned early at the age of 6 wk [14]. Hence, the low fecal pH could be a sign for inadequate digestion in the rumen, especially of starch. As the concentration of starch was common for calve and heifer feed (concentrate 371 g/kg dry matter (DM); TMR 182 g/kg DM) the rumen might not have been developed sufficiently to digest a typical adult cow diet even at the age of 5 months.

### Maturation of N metabolism in growing calves

Nitrogen-containing metabolites in blood and urine indicate changes in the diet, endogenous protein metabolism, and in developing microbial metabolism in the rumen. Dietary effects on N-containing metabolites were most likely associated with changes in microbial metabolism. Urea-N was measured in plasma [8] and urine, while non-urea-N was measured only in urine. Creatinine is the only N-containing metabolite in urine which is derived solely from muscle protein break-down, thus of endogenous origin [26]. Early weaning resulted in a sudden increase in urinary creatinine and urea, which indicated a catabolic status. Due to the change of highly digestible milk protein and energy to less digestible solid feed protein and energy, calves failed to adapt to weaning at early age. A fasting status was most likely established at the period of early weaning. Concomitantly, this was confirmed by a strong increase in plasma urea and beta-hydroxybutyrate and a low insulin concentration at early weaning [8] Furthermore, the quick rise in excretion of allantoin and uric acid, end products of metabolism of endogenous nucleic acids, might also be attributable to a catabolic state and/or a more stimulated microbial activity compared to late-weaned calves at this time. While the latter group showed a continuous increase of these compounds the calves weaned at experimental day 42 and changed to a TMR onwards, failed to further increase allantoin and uric acid concentration in urine. This might indicate a relative deficiency of energy available for adequate ruminal microbial protein synthesis due to a lower supply of easily fermentable substrates. Later, urinary urea concentrations decreased steadily in early-weaned calves, most likely due to recycling into the developing rumen to serve as N source for the microbial growth. Due to the prolonged high availability of milk protein and energy in late-weaned calves, urea concentrations in blood [8] and in urine were stable on a high level until weaning. At weaning, urea decreased by recycling into the rumen to compensate a lower dietary crude protein (CP) supply.

The purines from rumen microbes were metabolized and the end products hypoxanthine, xanthine, uric acid and allantoin were excreted in the urine [27]. Xanthine and hypoxanthine were only found in small amounts in cows’ urine, whereas uric acid and allantoin were the abundant purine derivatives [28]. This is due to the fact that, xanthine and hypoxanthine are converted to uric acid, which is further converted to allantoin [27]. Therefore, urinary concentrations of allantoin and its precursor, uric acid, were used as a predictor for microbial CP production in the rumen [29]. Both increased in early-weaned calves during weaning, but as they decreased again until the end of trial, their importance as a marker for rumen microbial activity can be contested. This has already been refuted for cows in different stages of lactation [29]. As stated above, the steep initial increase in allantoin and uric acid might be provoked by endogenous catabolic processes and only to a small extent by the increase in microbial activity at early weaning. Late-weaned calves expressed steady increases in urinary allantoin and uric acid concentrations, which slightly peaked around weaning. Therefore, it can be assessed that provision of liquid feed together with voluntary solid feed intake promoted a slow but effective rumen development and microbial activity. However, for the full functioning of rumen, the capacity for fibre digestion must be developed. For that, hippuric acid is a well-known marker [30]. It is formed in the liver from benzoic acid and glycine. Benzoic acid is synthesised by microbial metabolism of plant-derived phenolic cinnamic acids in the rumen [31]. Therefore, urinary hippuric acid concentration was strongly linked to diet composition in adult cows [32, 33], which seemed to be the same in calves as urinary hippuric acid concentration increased after weaning in both groups. Early-weaned calves, however, did not reach mature hippuric acid concentrations before experimental d 70, which could indicate an insufficient fibre digestion in the rumen, although they were on solid feed from d 42 on. Concomitantly, time spent chewing was increased only after experimental d 70, supporting the assumption that fibre digestion was not developed after early weaning and fibre intake was low, respectively. The potential restriction was reflected in a lower live weight gain and growth rates after early-weaning [8].

In addition, urinary N is a source of N_2_O emission [26], which affects the environment and can influence surface water, biodiversity and climate change in a negative way [34]. Therefore, attempts should be pursued in optimal rearing strategies to lower the excretion of urinary N-containing compounds.

### Rumen maturity at the age of 5 months

All variables linked to rumination, such as chewing behavior, rumen fill and sound, increased over time in both groups, despite the high MR allowance. These variables as well as VFA concentration and pH of ruminal fluid were not significantly different between the weaning groups at the end of the trial (d 140), which indicated that both groups reached a concordant status of rumen development. Therefore, rumen and its functions appeared to be equally developed at the age of 5 months, which corresponds to experimental d 140. Late-weaned calves showed no sign of impaired rumen development at that age despite of the higher weaning age. Ad libitum intake of MR in the first 5 wk of life did not retard rumen development [35]. High MR allowance and higher weaning age did not avert the transition to a functional ruminant status. Additionally, this transition was smoother in late-weaned calves [8]. On the contrary, in early-weaned calves these metabolic changes occurred relatively abrupt, which might have put a lot more strain on organs and tissues. In mammals, development of organs and tissues and their physiological functions in adulthood can be affected by early nutrition [36, 37]. As mentioned before, metabolic imprinting might occur through nutritional experiences in early life and can have a great impact on later health and performance [1]. Therefore, these abrupt changes in nutrition and consequent metabolic adaptations might lead to future impairment of health and performance. Increased MR intake by calves resulted in greater relative kidney weights [38, 39]. Hence, kidney development seemed to be affected by early-life nutrition. The secretion of protons and the rapid change of urinary composition during early-weaning might strain the calves’ kidney massively. This might also result in coping mechanisms that imprint these vital organs and alter their functions later in life. Although there was no difference in the measured variables at the age of 5 months, early-weaning might have caused a metabolic imprinting which might result in negative outcomes later in life. This might be true for calves born to primiparous mothers as well [40], even though we did not observe a considerable effect of parity on measured parameters. To assess the metabolic imprinting, these animals were further monitored in an ongoing observational study.

## Conclusion

Weaning calves at 7 wk of age appears to be too early for an adequate rumen development according to the evolutionary blueprint. This became apparent in an inefficient rumen function and a not fully developed metabolic adaption in early-weaned calves before experimental d 70, although they were fully weaned with 42 d, corresponded to an age of 7 wk. This indicated that calves needed more time to mature. They reached functional ruminant status at about 11 wk of age, which might be a more appropriate age for weaning. None of the response variables of behavior, rumen development, and urinary excretion of N-containing compounds were significantly different at the end of the trial. This indicated an equal functional status of the rumen despite the prolonged MR feeding in late-weaned calves. However, long-lasting effects of a metabolic imprinting for later life in terms of health and performance are possible for these future dairy cows and need to be studied.

## Material and methods

In accordance with the German Animal Welfare Act and approved by the Lower Saxony State Office for Consumer Protection and Food Safety (LAVES), Oldenburg, Germany, the present trial was carried out at the experimental station of the Institute of Animal Nutrition, Friedrich-Loeffler-Institute (FLI), Brunswick, Germany (file No.: 33.19–42,502-04–15/1858).

### Animals, housing, and diets

Detailed information about the study design were described elsewhere [8]. In short, female German Holstein calves (*n* = 59) were studied from the age of (mean ± standard deviation) 8 ± 2 days (d) until d 149 ± 2 of life. All calves originated from one established herd of German Holstein cows and were born within a seasonal calving period of 3 months (October - December). After the feeding trial, the animals remained in the institute´s own herd. They received 3 L of colostrum through a nipple bucket within 2 h after birth. Consistent quality of colostrum was evaluated using a colostrum densimeter (Wahl GmbH, Dietmannsried, Germany) and had to be defined as good at a value greater than 1035 g/L. In the pre-experimental feeding period, milk replacer (MR; NOLAC GmbH, Zeven, Germany) was mixed to the pooled herd milk, starting at the age of 3 d, with gradually increasing amounts from 0.30 kg MR powder/d (d 3 after birth) to 0.90 kg MR/d (d 5 after birth), when the maximum of 6 L liquid feed with a concentration of 150 g/L MR powder was available. Calves entered the study at an average live weight of 44.5 ± 5.2 kg at experimental d 1 and were moved into straw-bedded stables with provision of MR and concentrate through self-feeding systems (Förster-Technik GmbH, Engen, Germany). They were randomly allocated to either early weaning at 7 wk of age (early-weaned calves from multiparous cows (earlyMC, n = 16) and primiparous cows (earlyPC, *n* = 15)) or late weaning at 17 wk of age (late-weaned calves from multiparous cows (lateMC, *n* = 16) and primiparous cows (latePC, *n* = 12)) group assuring an equal allocation of calves from primiparous cows (PC, *n* = 17) and calves from multiparous cows (MC, *n* = 32). Early weaning was conducted at the age of 7 weeks, as this is a common management decision take on dairy farms, while late weaning was executed at the age of 17 weeks, because the reticulorumen volume approximately reaches adult proportions [3]. The trial started with 0.90 kg MR powder/d, which were available for all calves for the first 5 experimental d. Then MR was increased gradually within the next 5 d to 1.35 kg MR powder/d (9 L liquid feed) and remained at this level until the beginning of the weaning period (early-weaned group = experimental d 28; late-weaned group = experimental d 98). Over the entire trial, all calves received hay and water for ad libitum consumption. They had access to a maximum of 2 kg concentrate feed per d, which was reduced with start of late weaning (experimental d 98) to 1 kg/d. During weaning, the MR was reduced step-down within 14 d from 1.35 kg/d to 0.30 kg/d. Post-weaning calves were moved to another barn and received hay and a total mixed ration (TMR) consisting of 48% grass, 32% maize silage and 20% concentrate for ad libitum consumption.

### Behavioral assessments and measures of rumen variables

Calf behavior was recorded using instantaneous scan sampling ([41] at 10 min intervals during 2 h (h) once a wk between 8:00 and 12:00 h. Observation was done in a quiet atmosphere and the observer entered the barn 10 min before beginning to let calves accommodate themselves to the situation. As calves were raised on the farm, all typical activities and sounds outside the barn were accepted as characteristics for their d-to-d life and did not influence observation. Lateral movement of the mandibular in a lying position with a raised head was defined as chewing. Regurgitated boli were not included in the definition as they were difficult to observe. Therefore, chewing included both non-ruminating and ruminating activities. Lying on the ground with bent legs without movement of the mandibular was defined as resting. All other behaviors were registered as active behavior. To assess the percentage of each behavior during a given observational period, the number of observations of that defined behavior was divided by the total of 12 observations and multiplied with 100. For analysis, a given sampling d was combined as that d ± 3 d.

Rumen motility was assessed by counting the number of rumen sounds within 2 min with a stethoscope in the *Fossa paralumbalis*. Rumen fill was scored through palpation of the *Fossa paralumbalis* using a scale from 1 to 5, which was modified based on Zaaijer and Noordhuizen [42] (score 1 = empty, rectangular; score 2 = empty, triangular; score 3 = a little sunken, soft; score 4 = scarce blending between *Fossa paralumbalis* and *Arcus costalis,* firm; score 5 = *Arcus costalis* hardly visible, *Fossa paralumbalis* convex, firm). Scoring and measuring of rumen sounds were done on experimental d 1, 7, 14, 28, 42, 56, 70, 84, 98, 112, 126 and 140.

### Collection and analysis of blood, urine, saliva, rumen fluid and fecal samples

All samples were collected between 8 AM and 1 PM. Feces and urine were collected after spontaneous release or manual stimulation and filled in clean and sealable tubes. The pH value in feces and urine was measured 1 to 4 h after collection with a pH meter (pH 7119, WTW, Weilheim, Germany). For collection of saliva, two sterile cotton wool swabs were offered each animal on a clamp for chewing. Afterwards they were put into a commercially available salivette (Sarstedt, Nümbrecht, Germany) and cooled on ice. Salivettes were centrifuged at 2000 × *g* for 3 min and the pH value was measured with a pH meter (pH 7119, WTW, Weilheim, Germany). Urine was stored at -20 °C before analysis of net acid base excretion (NABE), urea, allantoin, creatinine, hippuric acid and uric acid were done. NABE and the base–acid ratio (BAR) were determined by the titrimetric method as described by Kutas [43]. Following dilution and filtration of urine samples, the purine derivates (allantoin, uric acid, hippuric acid) and creatinine were conducted by reverse-phase HPLC (LC-20A prominence, Shimadzu Europe GmbH, Duisburg, Germany) according to the method of Shingfield and Offer [44]. Urinary urea was analyzed with a colorimetric assay kit (Biovision, Milpitas, CA). Specific density of urine was estimated with a handheld refractometer (RPI, Optech srl, Turin, Italy). The pH values in whole blood were measured by an automated blood gas analyser (GEM4000, Werfen GmbH, Munich, Germany). On experimental d 140 ruminal fluid was collected using a rubber tube modified to Geishauser [45]. The equipment consisted of an oro-ruminal probe with flexible tube and a manual suction pump. The probe was inserted orally into the ventral sac of the rumen and a volume of approximately 100 ml of rumen fluid was collected after discarded the first part of the sample to avoid contamination by salvia. Ruminal pH was immediately determined using a pH meter (pH 7119, WTW, Weilheim, Germany). Ruminal fluid was centrifuged (Heraeus Varifuge®, 2400 × g, 5 min), acidified with a mixture of phosphoric acid/formic acid (5%) and stored at -20 °C before analysis of VFA (acetate, propionate, butyrate, iso-butyrate, valerate, iso-valerate) using a gas chromatograph (Clarus 689, PerkinElmer LAS GmbH, Rodgau, Germany) equipped with a flame ionisation detector as described by Geissler et al. [46]. Ruminal ammonia-N was determined directly after centrifugation using stream distillation according to DIN38406, E5-2 [47].

### Statistical Analysis

The pH value in feces and saliva was measured in two samples per animal and point in time, and the mean value was used for further analyses. NABE and BAR of the urine were calculated as:NABE = base excess – (acid excess + ammonium ions) BAR = sum of bases/sum of acids.

All variables, except for variables in ruminal fluid, were evaluated as repeated measures using PROC MIXED procedure in SAS (V 9.4., SAS Institute Inc., Cary, NC). The model included fixed factors of time, weaning age, parity of the mother and their interactions. Results were presented as least squares means (LSMeans) with standard errors (SE). All pair-wise differences of LSMeans were tested with the Tukey–Kramer procedure. Variables in ruminal fluid were evaluated by two-way ANOVA with weaning age, mother’s parity and their interaction as independent variables using SAS (V 9.4., SAS Institute Inc., Cary, NC). For all statistical tests, *p* < 0.05 was the level of significance. Visualization was done using GraphPad Prism 6.0 (GraphPad software, San Diego, USA), whereby measurements on serial time points were interpolated linearly.

## Data Availability

The datasets generated and analysed during the current study are available from the corresponding author on reasonable request.

## Abbreviations

ADF: Acid detergent fibre
BAR: Base–acid ratio
BW: Body weight
CF: Crude fiber
CP: Crude protein
d: Day
DM: Dry matter
earlyMC: Early-weaned calves from multiparous cows
earlyPC: Early-weaned calves from primiparous cows
EE: Ether extract
g: Gram
FLI: Friedrich-Loeffler Institut
h: Hour
HPLC: High performance liquid chromatography
kg: Kilogram
L: Litre
lateMC: Late-weaned calves from multiparous cows
latePC: Late-weaned calves from primiparous cows
LAVES: Lower Saxony State Office for Consumer Protection and Food Safety
LSMeans: Least squares means
mg: Milligrams
mmol: Millimoles
MC: Multiparous cows
Mo: Months
MR: Milk replacer
N: Nitrogen
NABE: Net acid base excretion
NDF: Neutral detergent fibre
P: Parity
PC: Primiparous cows
SE: Standard errors
SD: Standard deviation
T: Time
TMR: Total mixed ration
VFA: Volatile fatty acids
W: Weaning age
wk: Week
